# Effects of Combined Elicitors on Tanshinone Metabolic Profiling and *SmCPS* Expression in *Salvia miltiorrhiza* Hairy Root Cultures

**DOI:** 10.3390/molecules18077473

**Published:** 2013-06-27

**Authors:** Qiqing Cheng, Yunfei He, Geng Li, Yujia Liu, Wei Gao, Luqi Huang

**Affiliations:** 1 School of Traditional Chinese Medicine, Capital Medical University, Beijing 100069, China; 2 National Resource Center for Chinese Materia Medica, China Academy of Chinese Medical Sciences, Beijing 100700, China; 3 Protection and Utilization of Traditional Chinese Medicine of Beijing Area Major Laboratory, Beijing Normal University, Beijing 100875, China

**Keywords:** *Salvia miltiorrhiza*, hairy root, combined elicitors, SmCPS, tanshinones

## Abstract

Tanshinones are abietane-type norditerpenoid quinone natural products found in a well-known traditional Chinese medicinal herb, *Salvia miltiorrhiza* Bunge. The copalyl diphosphate synthase of *S. miltiorrhiza* (SmCPS) is the key enzyme in the first step for transformation of geranylgeranyl diphosphate (GGPP) into miltiradiene, which has recently been identified as the precursor of tanshinones. Based on previous gene-to-metabolite network, this study examined the influences of various combined elicitors on the expression of *SmCPS* and production of tanshinones in *S. miltiorrhiza* hairy root cultures. Combined elicitors were composed of three classes of elicitors, a heavy metal ion (Ag^+^), a polysaccharide (yeast extract, YE), and a plant response-signalling compound (methyl jasmonate, MJ). YE + Ag^+^, Ag^+^ + MJ, YE + MJ, and YE + Ag^+^ + MJ were the combinations we tested. The effect of elicitors on the *SmCPS* expression level was detected by quantitative real-time PCR (qRT-PCR), and the tanshinones accumulation responses to elicitation were analysed by Ultra Performance Liquid Chromatography (UPLC) metabolite profiling. Of these combined elicitors, the expression of *SmCPS* was significantly enhanced by elicitation, especially at 24 h and 36 h. Of four tanshinones detected, the contents of cryptotanshinone and dihydrotanshinone I were enhanced by treatment with YE + Ag^+^, Ag^+^ + MJ, and YE + Ag^+^ + MJ. Our results indicate that appropriate combined elicitors can enhance tanshinones production in hairy root cultures.

## 1. Introduction

**Figure 1:**
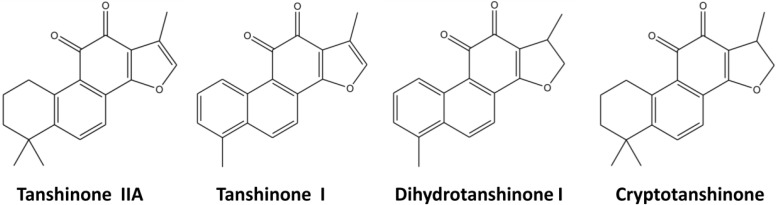
Major tanshinones in *S. miltiorrhiza*.

Tanshinones are abietane-type norditerpenoid quinone natural products found in *Salvia miltiorrhiza* Bunge (Dan shen in Chinese). The radix of *Salvia miltiorrhiza* is widely used in Chinese traditional medicine for the clinical treatment of many cardiovascular diseases such as blood circulation disturbances, inflammation and angina pectoris [1,2]. Tanshinones, the major lipophilic constituents of *Salvia miltiorrhiza*, show a variety of pharmacological activities including antibacterial, antioxidant, antiinflammatory and antineoplastic activities [3,4]. The more prevalent and more intensely studied tanshinones are tanshinone IIA, tanshinone I, dihydrotanshinone I, and cryptotanshinone (Figure 1).

Secondary metabolites of remarkable activities and complex structures found in plant tissues and cell suspension cultures have been extensively explored. To date, hairy root cultures, which are transformed plant roots by *Agrobacterium rhizogenes* carrying the Ri T-DNA plasmid, are preferred as biocatalyst over plant cell/callus and suspension cultures due to their genetic/biochemical stability, hormone-autotrophy, multi-enzyme biosynthetic potential mimicking that of the parent plants and relatively low-cost culture requirements [5]. As a result, these cultures have been used to produce the bioactive principles of several medicinal plants [6,7]. Moreover, *S. miltiorrhiza* hairy root culture has been shown to be a more efficient alternative to farm growth of whole plants for tanshinone production [8]. Elicitation can directly or indirectly induce plant secondary metabolite accumulation in plant tissues, cell cultures, and hairy root cultures. The strategy of elicitation works based on the accumulation of most secondary metabolites in plants is part of the defense responses of plants to pathogen infection and environmental stimuli, and elicitors were referred as the agents to induce plant defense responses [9]. Elicitation can also be utilized to identify genes involved in the biosynthesis of bioactive secondary metabolites [10,11]. In order to increase the quantity of the effective compounds, various elicitors have been studied with the hope of improving plant secondary metabolite [12]. *Silybum marianum* hairy root cultures treated with 2 mM Ag^+^ showed enhanced silymarin content (by up to 2 times) compared to non-treated hairy root cultures [13]. Ge *et al*. [14] examined the relationship between the accumulation of diterpenoid tanshinones and secondary metabolism pathways in *S. miltiorrhiza* hairy root cultures induced by YE and Ag^+^ treatment. The maximum enhancement of total tanshinones content was 1.2-fold with Ag^+^ and 3.1-fold with YE when compared to controls. In ginseng hairy root cultures, the levels of ginsenosides were enhanced after 7 days of MJ elicitation. Specifically, protopanaxadiol-type saponin and protopanaxatriol-type saponin contents were increased by 5.5–9.7 and 1.85–3.82 times the content of the control, respectively [15]. In conclusion, several classes of elicitors are generally used, which include heavy metal ions (Co^2+^, Ag^+^, and Cd^2+^), polysaccharides (yeast extract and chitosan), and plant response-signaling compounds (salicylic acid and methyl jasmonate). These elicitors can be used individually or in combination to stimulate the biosynthesis of target constituents. Zhao *et al*. [16] chose various elicitors and added them into the *Salvia miltiorrhiza* cell-culture system. The results indicated that Ag^+^, Cd^2+^ and YE were the most effective elicitors for stimulating production of tanshinones and combinations of elicitors had an additive or synergistic effect compared with that with a single elicitor on tanshinones accumulation.

In our recent study, we cloned the full-length cDNA of labdadienyl/copalyl diphosphate synthase (SmCPS) and performed functional analysis to find that SmCPS in *S. miltiorrhiza* is responsible for catalysing the protonation-initiated cyclization of GGPP to a bicyclic diphosphate intermediate. SmKSL can catalyse the ionization of diphosphate ester and the subsequent rearrangement reactions into miltiradiene, which is the key intermediate of the pharmacologically important tanshinone compounds [17,18]. SmCPS appears to be the first identified normal CPP specific CPS from an angiosperm that acts as the first class II diterpene cyclase to form the characteristic fused bicyclic hydrocarbon structure with particular stereospecificity [17]. In this study, we induced hairy root cultures from *S. miltiorrhiza* leaves by *A. rhizogenes* ACCC10060, and treated them with four combinations of elicitors, Ag^+^ + YE, Ag^+^ + MJ, YE + MJ, and Ag^+^ + YE + MJ. These combined elicitors were used to investigate the synergistic induction effect on *SmCPS* expression and the synthesis of tanshinones in hairy root cultures. Moreover, these studies provided more evidences for illustrating mechanisms of signal transduction following elicitation, as well as elicitors that enhance the production of secondary metabolites in plants.

## 2. Results and Discussion

### 2.1. Establishment of Salvia miltiorrhiza Hairy Root Cultures

After culturing on MS agar medium for 60 days, hairy root lines with *A. rhizogenes* successfully removed were transferred to 6,7-V liquid medium. Notably, hairy roots grew more rapidly in 6,7-V liquid medium than on MS agar medium. Hairy root culture is a promising system to provide high-value constituents [19], which has potential advantages over the cell and normal organ cultures for production of secondary metabolites, including genetic stability because of a higher degree of differentiation and organization than plant cell cultures, faster growth rate than normal plant organs in culture, and low maintenance in phytohormone-free media [7,20].

### 2.2. Effects of Combined Elicitors on SmCPS Expression

**Figure 2:**
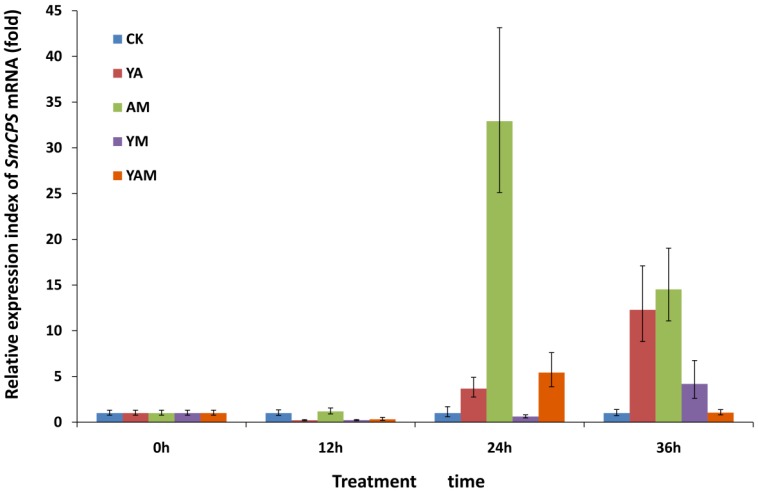
The expression levels of *SmCPS* of different treatments at different time. CK, the control hairy roots; YA, AM, YM and YAM, the hairy roots induced by YE + Ag^+^, Ag^+^ +MJ, YE + MJ and YE + Ag^+^ + MJ combined elicitors respectively. Data are means ± SE, n = 3.

The relative expression of *SmCPS* was detected by quantitative real-time PCR (qRT-PCR). The cDNAs of hairy roots treated with combined elicitors at different time points were chosen as templates. qRT-PCR analysis revealed that *SmCPS* expression was regulated through elicitation (Figure 2). For combined elicitors, both YE + Ag^+^ (YA) and YE + MJ (YM) treatments led to a gradual increase in *SmCPS* expression over time, which was most significant at 36 h and reached 12.3-fold and 4.3-fold of the expression level in the CK group, respectively. The Ag^+^ + MJ (AM) group showed a gradual increase during the first 24 h and the expression level was 32.9-fold greater than that in the CK group, which exhibited the most significant change for all groups. However, the expression in the group was reduced at the 36 h time point, and was 14.5-fold of the expression level in the CK group. The result of YE + Ag^+^ + MJ (YAM) treatment was similar to that of the AM group. The expression level increased initially and then reduced. The maximum level was 5.4-fold of the level in the CK group at 24 h while the expression level at 36 h was similar to that at the initial time point.

SmCPS is the first-step synthase for the transformation of GGPP into miltiradiene which has been proved to be the key intermediate of tanshinone compounds. It is worth noting that HMGR and DXR, the respective upstream enzymes of the MVA and MEP pathways, have been identified in *S. miltiorrhiza* hairy root. Their activities were proved to be stimulated by the biotic elicitor YE and the abiotic elicitor Ag^+^ [14]. It is also reported that 12 of the known tanshinone biosynthesis-related genes were evaluated by expression profiles after treated with YE, MJ, Ag^+^ and YE + Ag^+^, *SmHMGR*, *SmDXS2*, *SmFPPS*, *SmGGPPS* and *SmCPS* responsive to elicitation were identified as the potential key enzymes in the pathway for metabolic engineering to increase accumulation of tanshinone in *S. miltiorrhiza* hairy roots [21]. This indicated that proper elicitors might induce multiple enzymes in the pathway. In this study, the relative expression of *SmCPS* was regarded as the standard to screen the proper elicitor combinations. Since SmCPS was identified to play an important role in tanshinone biosynthesis, combined elicitors dramatically improved *SmCPS* expression and therefore could potentially be used as a synthetic inducer of tanshinone production.

### 2.3. Tanshinone Production in S. miltiorrhiza Hairy Root Cultures

**Figure 3:**
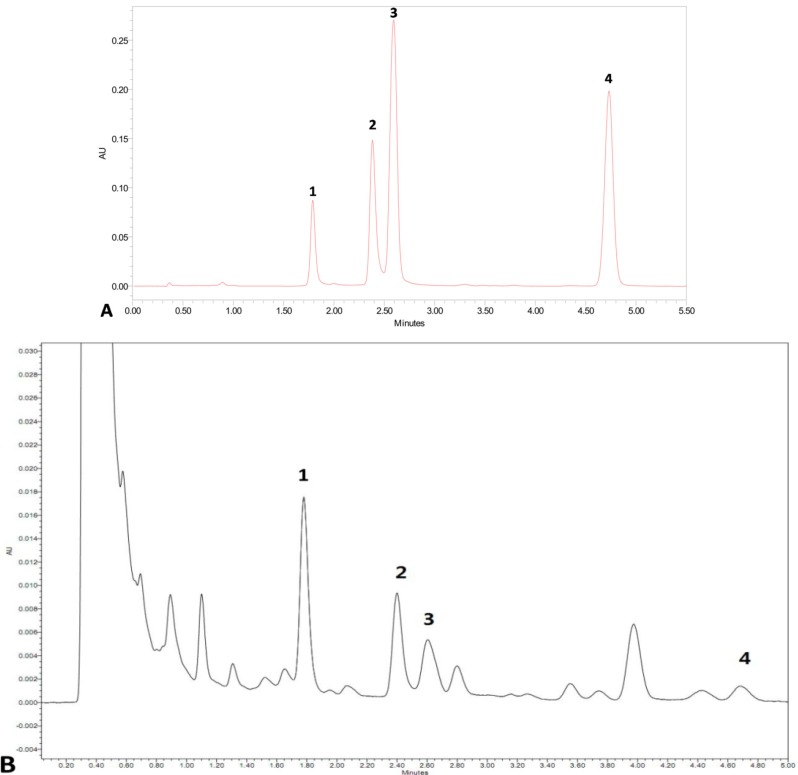
UPLC chromatograms of tanshinones from (A) mixture of authentic standards of tanshinones, and (B) combined elicitors treated hairy root cultures. 1 is dihydrotanshinone I, 2 is tanshinone I, 3 is cryptotanshinone, and 4 is tanshinone IIA.

Elicitors were added to hairy roots after 18 days in culture, and the hairy roots were harvested after 120 h. Since cryptotanshinone, dihydrotanshinone I, tanshinone I and tanshinone IIA are the major tanshinones in *S. miltiorrhiza*, we assayed for the production of these four tanshinones in hairy roots. Analysis of UPLC (Figure 3) indicated that cryptotanshinone, dihydrotanshinone I, tanshinone I and tanshinone IIA could be separated and then monitored. Retention time of dihydrotanshinone I was 1.8 min, that of tanshinone I was 2.4 min, that of cryptotanshinone was 2.6 min, and that of tanshinone IIA was 4.7 min (Figure 3). Detection of all four tanshinones was completed within 5 min. Our data indicate that analysis of these compounds is a rapid and accurate way to determine tanshinone content.

**Figure 4:**
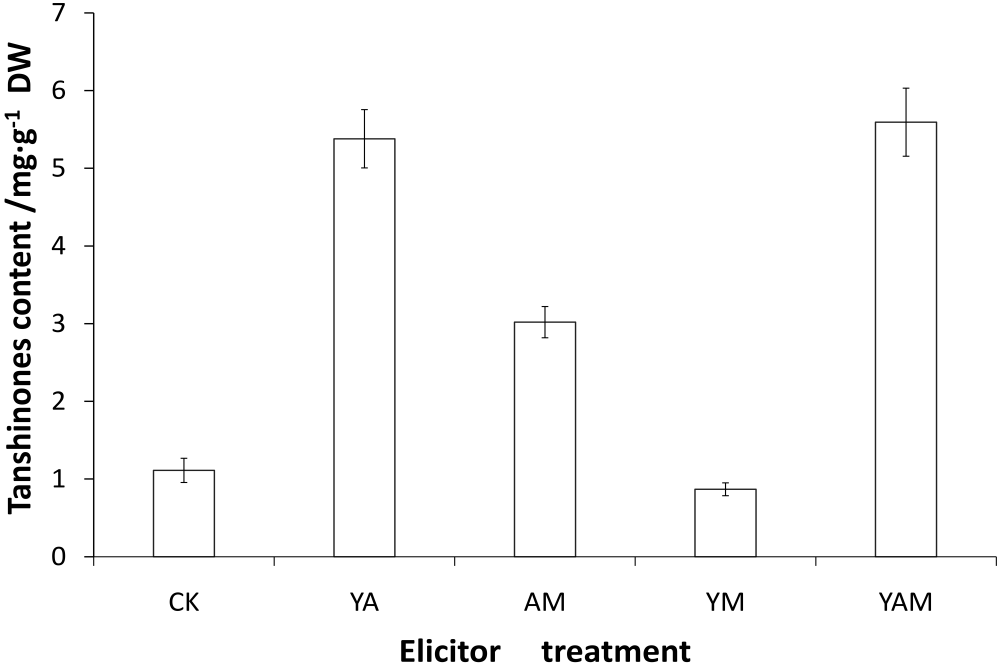
Effects of combined elicitors on total tanshinones accumulation of *S. miltiorrhiza* hairy roots (elicitors added to hairy roots on day 18 and hairy roots harvested after 120h); error bars for standard deviations, n = 3.

As shown in Figure 4, detailed analysis of the total content of tanshinones in hairy root cultures by different elicitation revealed low quantities of tanshinones in non-treated hairy roots. Our results show that treatments with YE + Ag^+^ and YE + Ag^+^ + MJ enhanced the level of total tanshinones up to nearly five times that of the control group. Thus, elicitation was a potent tool to regulate the accumulation of tanshinones. Appropriate elicitation is observed as an auxiliary method for the biosynthesis of secondary metabolites.

However, the content of tanshinones was only 2.7-fold that of the control group after treatment with Ag^+^ + MJ. There was even no obvious change in tanshinones after treatment with YE + MJ. It is thus suggested that not all classes of elicitors in combination have a synergistic effect on accumulation of secondary metabolites. In our study, YE + Ag^+^ and YE +Ag^+^ + MJ were the most effective combined elicitors for stimulating tanshinone accumulation. As reported previously, combined elicitors were more effective than single elicitors on tanshinone accumulation [9,16]. Combined elicitors including Ag^+^ enhanced the biosynthesis of tanshinones to levels greater than that achieved by combined elicitors consist of YE and MJ, so treatment with elicitors combined with the heavy metal ion Ag^+^ might be effective for tanshinone production.

### 2.4. Effects of Combined Elicitors on the Accumulation of Cryptotanshinone and Dihydrotanshinone I

**Figure 5:**
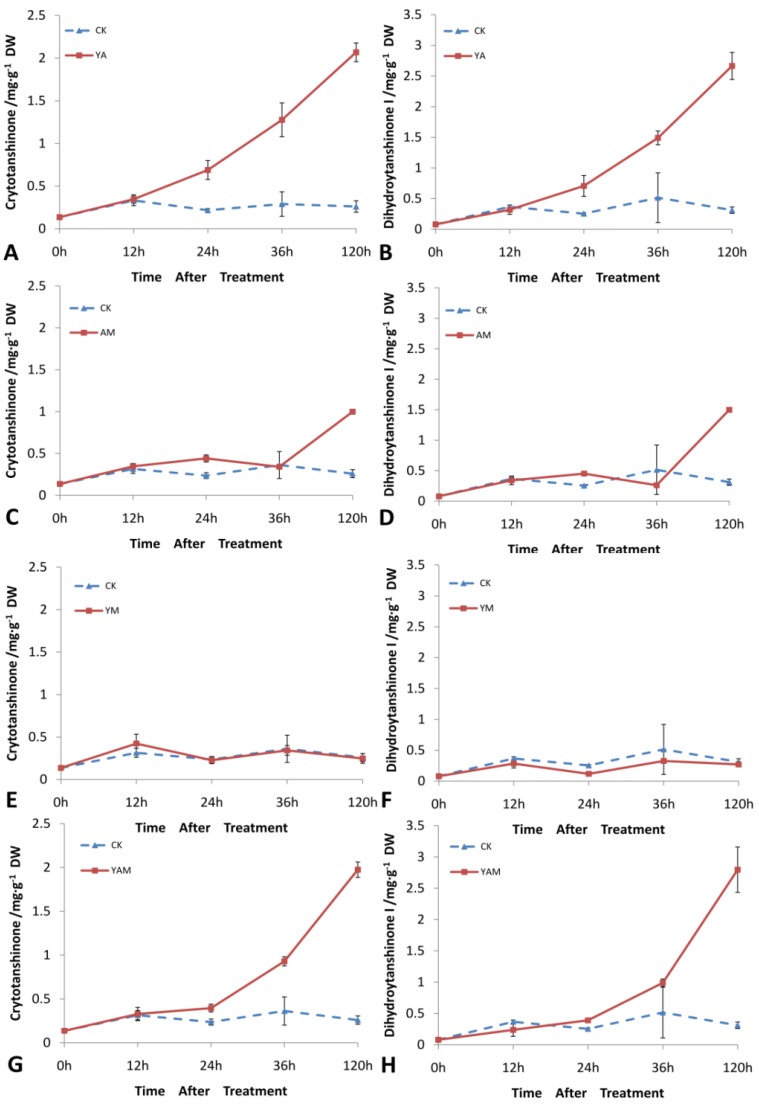
Time courses of cryptotanshinone and dihydrotanshinone I contents in *S. miltiorrhiza* hairy root cultures after treatment with YA (YE + Ag^+^), AM (Ag^+^ + MJ), YM (YE + MJ) and YAM (YE + Ag^+^ + MJ); error bars for standard deviations, n = 3.

The four types of tanshinone compounds detected by UPLC were cryptotanshinone, dihydrotanshinone I, tanshinone I, and tanshinone IIA. Cryptotanshinone and dihydrotanshinone I were clearly stimulated by elicitation, but the quantities of tanshinone I and tanshinone IIA didn’t differ dramatically. Accumulation of tanshinones after elicitors treatments were as follows (Figure 5).

The accumulation levels did not significantly change after YM treatment. YA treatment exponentially increased the accumulation of dihydrotanshinone I and cryptotanshinone from 0 h to 120 h, and the maximal content of dihydrotanshinone I and cryptotanshinone was 8.6-fold and 8.0-fold that of the CK group. The changes in the accumulation level with AM treatment were that dihydrotanshinone I gradually increased and reached a maximum of 4.8-fold at 120 h when compared to the level in the CK group, whereas cryptotanshinone was 3.9-fold that of the CK group. The changes in the accumulation level with YAM treatment was that dihydrotanshinone I and cryptotanshinone increased slowly until 24 h, and from 36 h to 120 h, they rapidly increased, dihydrotanshinone I and cryptotanshinone were 9.0-fold and 7.6-fold greater than the levels in the CK group, respectively.

In the *S. miltiorrhiza* culture systems, cryptotanshinone was the major tanshinone species stimulated by various elicitor treatments. Cryptotanshinone has been identified as a phytoalexin in *S. miltiorrhiza* plant and is involved in the passive and active plant defense response to pathogens [22]. The accumulation of cryptotanshinone could be stimulated by elicitors in *S. miltiorrhiza* cell and hairy root cultures [23,24], which might act as a defense or stress response. Zhang *et al*. [25] found that cryptotanshinone and dihydrotanshinone I were promoted by elicitation. This suggests that dihydrotanshinone I accumulation may work the same way as cryptotanshinone, or they may transform to each other by isomerization or biosynthesis, which more work needs to be done to find out.

YE + MJ treatment showed an insignificant and repressive effect on tanshinone accumulation in *S. miltiorrhiza* hairy root cultures. Although MJ stimulates plenty of secondary metabolites in medical plant cultures [26,27], the effect of MJ on the accumulation of tanshinones is not obvious, especially in the initial stages of treatment. This discrepancy suggests that the effects of various elicitors on secondary metabolite production in plant tissue cultures depend on the specific secondary metabolites [16]. Alternatively, MJ may stimulate tanshinones in the later stage of elicitors treatment, Kai *et al*. [21] reported that the tanshinones content was enhanced by treated with MJ after 6 days. In previous studies, the YE elicitor was observed as a potent elicitor for stimulating the tanshinone production in cultures of *S. miltiorrhiza* [23,24,25]. This indicates that elicitors in combination may also have an antergic effect. It is also possible that the time of tanshinones accumulation by YE + MJ elicitors is beyond our treatment time range, so the effect of elicitation on the secondary metabolite production may depend on the target compound species, the dosage and combination of elicitors, and time range after elicitor addition. Ag^+^ has been applied to many hairy root cultures of diverse plant species, and it has a universal effect on promoting the content of secondary metabolites. In our study, combined elicitors contain Ag^+^ enhanced the accumulation of dihydrotanshinone I and cryptotanshinone more than that achieved by YE + MJ, so elicitors combined with Ag^+^ may act as the effective combination to improve the accumulation of dihydrotanshinone I and cryptotanshinone.

It has been reported that plant secondary metabolism is most often regulated at the transcription level [28]. The observed induction by elicitors and enhancement in *SmCPS* mRNA level prior to tanshinones accumulation were similar to previous findings that in rice transcriptional up-regulation prior to rice labdane-related diterpenoid phytoalexin accumulation [17,29]. Therefore, the *SmCPS* expression level and tanshinones accumulation were regarded as standards to investigate elicitors. In order to clone key enzymes with low expression, mimic potential multi-enzyme biosynthesis pathway of the parent plants, or mediate biotransformation of exogenous substrates, the treatment time point with high mRNA expression level is preferred. To improve the tanshinone accumulation or to screen the optimal culture conditions, the treatment time point with high tanshinones amount was selected. So that the choice of treatment time points depends on the purposes.

Additionally, more work is needed to be done to clone the full-length cDNAs and identify the functions of specific enzymes in downstream tanshinone biosynthesis pathway, such as cytochrome P450. This will do a great favor to discover the unknown functional mechanism why elicitor in combinations can result in synergistic effect of tanshinone accumulation. As more studies to be conducted, it is possible to optimize the culture condition to produce tanshinones in laboratory or in industry at large scale.

## 3. Experimental

### 3.1. Plant Materials

Seeds of *Salvia miltiorrhiza* Bunge were surface sterilized by 0.1% mercuric chloride and cultured on hormone-free MS agar medium (Murashige and Skoog, 1962) [30]. The MS medium contained 30 g L^−^^1^ sucrose and 7 g L^−1^ agar for germination. Cultures were maintained at 25 °C under a 16 h light/8 h dark photoperiod in a growth chamber fitted with a cool white-fluorescent lamp providing a PPFD level of 25 μmol m^−^^2^ s^−^^1^.

### 3.2. Growth of Agrobacterium rhizogenes and Hairy Root Cultures

The culture of *A. rhizogenes* strain ACCC10060 was initiated from glycerol stock and grown overnight at 28 °C with shaking (200 rpm) in liquid YEB medium to mid-log phase (OD_600_ = 0.8). Excised leaves of *S. miltiorrhiza* were pre-cultured in MS agar medium for 2 days at 25 °C with a 16 h light/8 h dark photoperiod. The leaves were dipped into *A. rhizogenes* ACCC10060 liquid inoculation medium for 10 min to infect leaf explants, blotted dry on sterile filter paper, and incubated in dark at 25 °C on MS agar medium containing 400 mg L^−1^ cefotaxime (cef). After co-cultivation for 2 days, leaf explants were transferred to a hormone-free MS agar medium for hairy roots induction. Numerous hairy roots were observed emerging from the wound sites within 2 weeks. After the hairy roots were 3–5 cm, they were separated from the explants and cultured in dark at 25 °C on MS agar medium with gradual decrease of cefotaxime interval of 10 days. After being repeated transferred to fresh medium, rapidly growing hairy root cultures were obtained. These isolated root clones were transferred to 50 mL of 6,7-V liquid medium in 100 mL flasks. Hairy root cultures were maintained at 25 °C on a gyratory shaker (80 rpm) in dark in a growth chamber.

### 3.3. Elicitation of Combined Elicitors

Combined elicitors of YE + Ag^+^, Ag^+^ + MJ, YE + MJ, and YE + Ag^+^ + MJ, were added to *S. miltiorrhiza* hairy root cultures. YE was the carbohydrate fraction of yeast extract prepared by ethanol precipitation, as described previously [14]. Briefly, yeast extract (25 g) was dissolved in distilled water (125 mL) and mixed with ethanol (100 mL). The solution was allowed to precipitate for 4 days at 4 °C in a refrigerator, and the supernatant was decanted. The gummy precipitate was redissolved in distilled water (125 mL) and subjected to another round of ethanol precipitation. The final precipitate was dissolved in distilled water (100 mL) and sterilized by autoclaving at 121 °C for 20 min. MJ was dissolved in absolute ethyl alcohol to the concentration of 200 mM and sterilized by filtering through a microfilter (0.22 µm). Ag^+^ was dissolved in distilled water to the concentration of 300 mM and sterilized by filtration (0.22µm membrane). These three mother solutions of elicitors were stored at 4 °C prior to use.

Elicitors were administered to the shake-flask culture of *S. miltiorrhiza* hairy roots on day 18. Each of the elicitor solutions was added into the culture medium at the desired concentration and combinations. The combinations were YE + Ag^+^, YE + MJ, MJ + Ag^+^, and YE+ Ag^+^ + MJ, and the final concentration of YE, MJ, and Ag^+^ were 2.5 mg mL^−^^1^, 200 µM, and 100 µM, respectively. After 12 h, 24 h, and 36 h treatment with different combined elicitors, hairy roots were collected for *SmCPS* expression analysis and determination of tanshinone content. And after 120 h the hairy roots were harvested for tanshinone content analysis. All treatments were performed in triplicate, and the results were averaged.

### 3.4. Metabolite Analysis of Hairy Roots

Harvested hairy roots (0.05 g) were powdered and extracted with methanol. The samples were filtered through a 0.22 µm microporous membrane and stored in a flask. The extracts were analysed by UPLC on an ACQUITY UPLC C_18_ reverse phase column (2.1 × 50 mm, 1.7 µm) at temperature 35 °C. The solvent gradient used in this study was formed through with proportion of mix of solvent A (0.5% methanoic acid in water) and solvent B (acetonitrile): 0~1.5 min 49% A; 1.5~5 min 49%~46% A; 5~5.1 min 46%~10% A; 5.1~5.8 min 10% A; 5.8~6 min 10%~49% A. The flow rate of the solvent was kept constant at 0.4 mL min^−1^. Samples (20 μL) were detected at wave lengths of 280 nm. Four tanshinone species cryptotanshinone, dihydrotanshinone I, tanshinone I and tanshinone IIA were detected and quantified using authentic standards obtained from the Institute for Identification of Pharmaceutical and Biological Products (Beijing, China). We identified the tanshinones by matching the retention times and spectral characteristics to those from single UPLC run of these four tanshinone standards. Tanshinones content is the total content of the four reported tanshinones in the hairy roots.

### 3.5. Quantitative Real-Time PCR

Total RNA was extracted from the harvested hairy roots by using the Trizol method (Invitrogen, Carlsbad, CA, USA). An aliquot (1 µg) of the total RNA was used and the first strand cDNA was synthesized from total RNA by using the reverse-transcription PCR system according to the manufacturer’s protocol of Primescript 1st Strand cDNA Synthesis Kit (Takara, Tokyo, Japan). The primer pairs used for these assays are shown in Table 1. qRT-PCR was performed according to the manufacturer’s instructions (Applied Biosystems, Foster City, CA, USA), and gene expression was quantified with the comparative C_T_ method (also known as the 2^−ΔΔC^^T^ method).

**Table 1:** Primers sequences of the target and housekeeping gene.

**gene**	**Sequences of the primers(5′→3′)**
***SmCPS***	GAGGGAGAGGTGAGGAAGGAA
	AGGGAACAAAAGTTGAAAAGG
***β-Actin***	AGGAACCACCGATCCAGACA
	GGTGCCCTGAGGTCCTGTT

### 3.6. Statistical Analysis

Each result shown in the figures indicates the mean of three replicate treatments. Data were expressed as the mean ± SD. A comparison of multiple groups was conducted using one- or two-way analysis of variance (ANOVA) analysis, and in all cases, the confidence coefficient was set at *p* < 0.05.

## 4. Conclusions

In conclusion, the relative expression level of *SmCPS* was stimulated by YE + Ag^+^, Ag^+^ + MJ, YE + Ag^+^ + MJ elicitors, and most strongly by Ag^+^ + MJ which enhanced it to more than 30 times that of the control group. The treatments of YE + Ag^+^, Ag^+^ + MJ, YE + Ag^+^ + MJ stimulated the tanshinone production in *S. miltiorrhiza* hairy root cultures, especially of cryptotanshinone and dihydrotanshinone I. The *SmCPS* mRNA expression level and tanshinone accumulations were regarded as standards to compare the elicitors. By using these elicitors based on previous studies, the hairy root cultures of *S. miltiorrhiza* are stable and effective systems for cryptotanshinone and dihydrotanshinone I production after elicitation. The effects of elicitation on tanshinone production may result from the species of target compounds, the dosage and combination of elicitors, and treatment time range. Meanwhile, different time points of elicitor treatment we choose depends on the experimental purposes. In order to clone key enzymes with low expression, mimic potential multi-enzyme biosynthesis pathway of the parent plants, or mediate biotransformation of exogenous substrates, the treatment time point with high mRNA expression levels of target gene was preferred. To improve the tanshinone production or to screen the optimal culture conditions, the treatment time point with high tanshinone quantities was selected. The results provide strong evidence for further studies on the mechanisms of elicitation and the signal pathways involved in tanshinone biosynthesis. Additionally, it shows the need to discover the unknown functional mechanism why elicitor in combinations can result in synergistic effect of tanshinone accumulation. With further studies to thoroughly investigate the elicitation mechanism, it could be possible to produce tanshinones in the laboratory or in industry at an even larger scale in a near future.
